# The orphan nuclear receptor *Nr4a1* mediates perinatal neuroinflammation in a murine model of preterm labor

**DOI:** 10.1038/s41419-019-2196-7

**Published:** 2020-01-06

**Authors:** Sarah M. Estrada, Andrew S. Thagard, Mary J. Dehart, Jennifer R. Damicis, Elisabeth M. Dornisch, Danielle L. Ippolito, Irina Burd, Peter G. Napolitano, Nicholas Ieronimakis

**Affiliations:** 1 Department of Obstetrics and Gynecology, Division of Maternal Fetal Medicine, Madigan Army Medical Center, Tacoma, WA USA; 2 Department of Clinical Investigation, Madigan Army Medical Center, Tacoma, WA USA; 3 PharmaWrite Medical Communications LLC, Princeton, NJ USA; 4 Integrated Research Center for Fetal Medicine, Johns Hopkins University School of Medicine, Baltimore, MD USA; 5 Department of Obstetrics and Gynecology, University of Washington Medical Center, Seattle, WA USA

**Keywords:** Disease model, Cellular neuroscience

## Abstract

Prematurity is associated with perinatal neuroinflammation and injury. Screening for genetic modulators in an LPS murine model of preterm birth revealed the upregulation of *Nr4a1*, an orphan nuclear transcription factor that is normally absent or limited in embryonic brains. Concurrently, *Nr4a1* was downregulated with magnesium sulfate (MgSO_4_) and betamethasone (BMTZ) treatments administered to LPS exposed dams. To understand the role of *Nr4a1* in perinatal brain injury, we compared the preterm neuroinflammatory response in *Nr4a1* knockout (KO) versus wild type (wt) mice. Key inflammatory factors *Il1b*, *Il6* and *Tnf*, and Iba1+ microglia were significantly lower in *Nr4a1* KO versus wt brains exposed to LPS in utero. Treatment with MgSO_4_/BMTZ mitigated the neuroinflammatory process in wt but not *Nr4a1* KO brains. These results correspond with a reduction in cerebral hemorrhage in wt but not mutant embryos from dams given MgSO_4_/BMTZ. Further analysis with *Nr4a1-GFP-Cre* × *tdTomato loxP* reporter mice revealed that the upregulation of *Nr4a1* with perinatal neuroinflammation occurs in the cerebral vasculature. Altogether, this study implicates *Nr4a1* in the developing vasculature as a potent mediator of neuroinflammatory brain injury that occurs with preterm birth. It is also possible that MgSO_4_/BMTZ mitigates this process by direct or indirect inhibition of *Nr4a1*.

## Introduction

Preterm delivery and the long-term impacts on the developing neonate remain a major concern for obstetric care in the United States. The rate of preterm birth in the United States continue to rise^1^. Though our understanding of preterm birth and the associated fetal complications continues to evolve, there is still much to address. The pathogenesis of preterm birth and its consequences is complex and multifactorial, likely resulting from numerous elements including intrauterine inflammation and dysregulation of fetal neurodevelopmental processes^2,3^.

Preterm delivery, particularly when earlier in gestation, is linked to a high prevalence of cognitive impairment, developmental delays, and central nervous system disorders, such as cerebral palsy (CP)^4,5^. Perinatal brain injury is thought to result from acute neuroinflammation, driven by an excess of cytokines and immunological responses^2,6^. Treatment aimed at reducing perinatal neuroinflammation includes magnesium sulfate (MgSO_4_), which is thought to reduce vascular instability, decrease pro-inflammatory cytokines, and/or prevent hypoxic injury and ischemia-induced tissue damage^6^. Although the underlying molecular mechanisms of MgSO_4_ remain poorly understood, antenatal use has been shown to decrease the incidence of CP. Several randomized controlled trials have demonstrated improved outcomes with MgSO_4_, particularly for preterm pregnancies treated before 32–34 weeks gestation^7–12^. However, the effectiveness of MgSO_4_ is not guaranteed with an estimated number needed to treat of 56^13^.

In conjunction with MgSO_4_, antenatal corticosteroids such as betamethasone (BMTZ), have also improved fetal outcomes. Trials using corticosteroids in preterm labor have demonstrated reductions in intracranial hemorrhage and cystic periventricular leukomalacia^14,15^. Corticosteroids are characteristically anti-inflammatory and may alleviate the fetal neuroinflammatory burden with prematurity. Analogous to MgSO_4_, the exact molecular mechanisms of corticosteroids remain unclear^16^. If the mechanisms of injury and the actions of these treatments are delineated, other interventions may be developed.

We sought to identify pro-inflammatory targets that are responsive to MgSO_4_ and BMTZ treatments. Using microarray screening, we identified the upregulation of *Nr4a1* in a murine model of preterm labor. *Nr4a1* is an orphan nuclear receptor, also known as Nur77, TR3, and NGFI-B, that has been implicated in a variety of immune responses and adult neuroinflammatory injury^17–20^. In the prenatal brain, *Nr4a1* expression is limited and not required for normal development^21–23^. Based on these preliminary findings, we hypothesized that *Nr4a1* plays an important role in perinatal brain injury and sought to characterize its role using mutant mouse models. Our primary objective was to evaluate the neuroinflammatory response of *Nr4a1* knockout (KO) versus wild type (wt) mice. Additionally, we sought to evaluate the relationship between *Nr4a1* KO and wt mice in conjunction with MgSO_4_ and BMTZ treatments. Finally, we used Cre-loxP fate mapping to identify the cellular expression of *Nr4a1* that is upregulated in response to perinatal neuroinflammation.

## Materials and methods

### Animal models

All animal experiments and procedures were approved by the Institutional Animal Care and Use Committee. Animals were randomly selected to receive treatments in all experiments/groups and the data acquisition/analysis was performed blinded with coded samples. For the microarray screening, CD-1 mice were purchased from Charles River Laboratories (Wilmington, MA, USA) and on E15.5 received an intrauterine injection of 100 µl containing 100 µg lipopolysaccharide (LPS) from *Escherichia coli* O55:B5 (Sigma-Aldrich, St. Louis, MO, USA) or the vehicle (PBS)^24^. Mice were subsequently treated with normal saline (NS) or MgSO_4_/BMTZ, 30 min post-injection of LPS/PBS. MgSO_4_ and BMTZ treatments were administered subcutaneously as previously described^25^. Brains were harvested 6 h following the intrauterine injections and frozen immediately in liquid nitrogen for expression analysis. Sample sizes were estimated based on cytokine gene expression from previous studies^26,27^.

*Nr4a1* KO and wt controls on a C57BL/6 background were acquired from The Jackson Laboratory (Bar Harbor, ME, USA)^22^. Once acclimated (2 weeks), males and females were paired by their respective genotypes: KO male with KO female and wt male with wt female. Pregnancy was confirmed by the presence of a vaginal plug at which time males were separated from the females. At E15.5, females received a 100 µl intrauterine injection of either the PBS vehicle or 250 µg of LPS. The higher LPS dose was chosen for this and subsequent experiments in order to evaluate a more severe neuroinflammatory response that has been reported for the C57BL/6 strain^28^. A second group of wt and *Nr4a1* KO mice received LPS or PBS and subsequent treatment with MgSO_4_/BMTZ as previously described. Embryos were again collected 6 h following the intrauterine injections. In these experiments, brains were harvested from half of the embryos for expression analysis while the remainder were placed in formalin, cut sagittal and embedded in paraffin.

For fate mapping, *Nr4a1-GFP-Cre* recombinase and *tdTomato loxP* reporter mice were acquired from The Jackson Laboratory; strains *C57BL/6-Tg(Nr4a1-EGFP/cre820Khog/J* and *B6.Cg-Gt(ROSA)26Sortm14(CAG-tdTomato)Hze/J* respectively. The *Nr4a1*-*GFP-Cre* allele enables the expression of green fluorescent protein (GFP) and Cre simultaneously under the *Nr4a1* promoter^29^. The *tdTomato loxP* allele (Ai14 variant) is irreversibly activated in the presence of Cre^30^. Offspring with both alleles were generated by mating *Nr4a1*-*GFP-Cre* males with *tdTomato loxP* females. Dams received a 100 µl intrauterine injection of either 250 µg LPS or the PBS vehicle. In these experiments, only LPS exposed mice received MgSO_4_/BMTZ. Six hours post-intrauterine injections, embryo brains were initially fixed by maternal cardiac perfusion. First, the right ventricle was punctured to permit drainage and then 10 ml PBS followed by 10 ml 4% formaldehyde were infused into the left ventricle^31^. Embryos were then placed in 5 ml 4% formaldehyde for an additional 2 h and then dehydrated with a sucrose gradient beginning with 10%, 20%, and then 30%, in which they were left overnight at 4 °C. The next day embryos were cut sagittal and frozen in optimal cutting temperature (OCT) compound.

### Microarray

#### RNA isolation

While working on dry ice, individual CD-1 embryo brains were selected and transferred to a pre-chilled 2.0 ml tube containing one 5 mm stainless steel bead (Qiagen, Germantown, MD, USA). Once brains were transferred, 700 μl of QIAzol Lysis Reagent (Qiagen) was added to each sample. Samples were immediately homogenized using a TissueLyzer LT (Qiagen) for 5 min at 50 Hz, then placed at −80 °C until all samples were processed. RNA was extracted using the miRNeasy 96 kit (Qiagen), according to the manufacturer’s instructions. The quantity of RNA samples was evaluated with a multichannel Nanodrop 8000 spectrophotometer (Thermo Fisher, Waltham, MA, USA).

#### Gene array

With an input of 100 ng total RNA, hybridization-ready, fragmented, labeled, sense-stranded DNA targets were prepared using Affymetrix GeneChip® WT Plus Reagent Kit (Affymetrix, Santa Clara, CA, USA). We then prepared the labeled cDNA targets, trays, and arrays with the GeneTitan Hybridization, Wash and Stain Kit for WT array plates (Affymetrix). Samples were then applied to the Mouse Gene 2.1 ST 96 Array Plate and placed in the GeneTitan System (Affymetrix). These steps conformed to the manufacturer’s recommendations. Of the 96 arrays, 80 passed the GeneTitan scanning quality control which includes visual inspection and QC metrics provided by the Affymetrix Expression Console (hybridization control performance, labeling control performance, internal control gene performance, signal histogram, probe cell intensity, Pearson’s Correlation, and Spearman Rank Correlation). In order to verify the performance of the replicates, Pearson’s R^2^ was manually calculated for each set of replicates within each group. The 16 which did not meet criteria were repeated. All 16 repeats passed quality control and were included in the final analysis.

#### Gene array data analysis

We normalized arrays using an extension of the *PLIER* (Probe Logarithmic Intensity Error) algorithm, called the iterPLIER procedure, in the Affymetrix Expression Console. The iterPLIER (gene level) procedure discards feature sets that perform poorly, as described by Qu et al.^32^. We imported the resulting CHP files into Partek Genomics Suite version 6.12.0907 (St. Louis, Missouri, USA). Affymetrix library files included all available reference files related to MoGene-2_1-st. To determine differentially expressed genes, an analysis of variance (ANOVA) was conducted with contrasts. Gene lists were generated by applying a cutoff by Benjamini-Hochberg False Discovery Rate (FDR < 0.05)^33,34^.

### qRT-PCR

E15.5 brains were snap frozen in liquid nitrogen and subsequently processed for gene expression analysis. Whole brains were homogenized and then RNA was extracted utilizing the RNeasy Lipid Tissue Kit (Qiagen), following the manufacturer instructions. First strand cDNA was then generated using the GoScript reverse transcription kit (Promega, Fitchburg, WI, USA). PCR reactions were conducted with SYBR Green on a Light Cycler 480 II system (both from Roche, Indianapolis, IN, USA). Reactions were performed in triplicate for each gene sample using the following conditions: 95 °C × 5 min, then 40 cycles at 95 °C × 10 s, 60 °C ×10 s, and 72 °C × 10 s. The relative expression of each respective target was calculated by ΔCt, normalizing to ribosomal protein *18* *s*^35,36^. Primer sequences for *Foxd1* were generated using NCBI primer-BLAST^37^. With exceptions to *18* *s* and *Foxd1*, the majority of primers sequences were generated by the Harvard PrimerBank^38^. Primer sequences are detailed in Supplementary Table S1.

### Histology

Sagittal sections from whole embryos placed in formalin or OCT were cut 8 µm thick. Formalin fixed paraffin embedded sections were cleared and used for Iba1 immunohistochemical staining. Antigen retrieval was performed using citrate buffer pH 6.0 (Vector Laboratories, Burlingame, CA, USA) for 25 min at 95 °C. Slides were blocked with biotin/streptavidin (Vector Laboratories) in accordance with the manufacturer’s instructions and then with 2.5% Horse serum (Vector Laboratories) for 1 h. Washes were performed with PBS + 0.05% Tween 20 and a polyclonal goat anti-Iba1 (Abcam ab107159, Burlingame, CA, USA) was added at 1:1000 overnight at 4 °C in PBS + 0.05% Tween 20 with 1% BSA. The next day, an anti-goat pre-diluted ready to use secondary (Vector Laboratories) was applied for 1 h, followed by DAB staining. Slides were counterstained with hematoxylin and Iba1+ cells were quantified within 20× fields of fetal brains using the ImageJ cell counter application. The mean number of Iba1+ cells was calculated relative to the dimensional area of our microscopes 20× objective (358 mm^2^). Paraffin slides were also cleared and stained with hematoxylin and eosin (H&E) to visualize pathology. The frequency of cerebral hemorrhage was counted, either present or absent from at least three fields for each brain^39^. Frozen slides were examined under fluorescence to identify GFP and tdTomato reporters. Sections were stained for GFP to enhance the signal^40^. For this a polyclonal chicken anti-GFP (Abcam ab13970) was added at 1:1000 for 1 h, followed by a donkey anti-chicken secondary conjugated to Alexa Fluor 488 (Jackson ImmunoResearch, West Grove, PA, USA), also at 1:1000 for 1 h. Tissues were counterstained with DAPI and biotinylated BS1 (Vector Laboratories) at 1:500 for 1 h and followed by streptavidin Alexa Fluor 647 (Jackson ImmunoResearch) at 1:1000 for 1 h. All images were captured on a Leica (Buffalo Grove, IL, USA) SP8 system with DFC7000 camera. The brightness and contrast for fluorescent images were adjusted evenly between experimental and control samples to reduce background.

### Statistics

With exception to the aforementioned microarray analysis, gene expression comparisons were accomplished with either unpaired Student’s *t*-test or one-way ANOVA with and without Tukey’s post-hoc test. Categorical data (Figs. 5, 6) was analyzed by chi-squared test using the PBS or PBS + MgSO_4_/BMTZ control groups as the expected reference. These comparisons were conducted using either Excel (Microsoft, Redmond, WA, USA) or SPSS (IBM, Armonk, NY, USA). Graphs and tables were generated using Excel.

## Results

### *Nr4a1* expression is upregulated with perinatal neuroinflammation

**Fig. 1:**
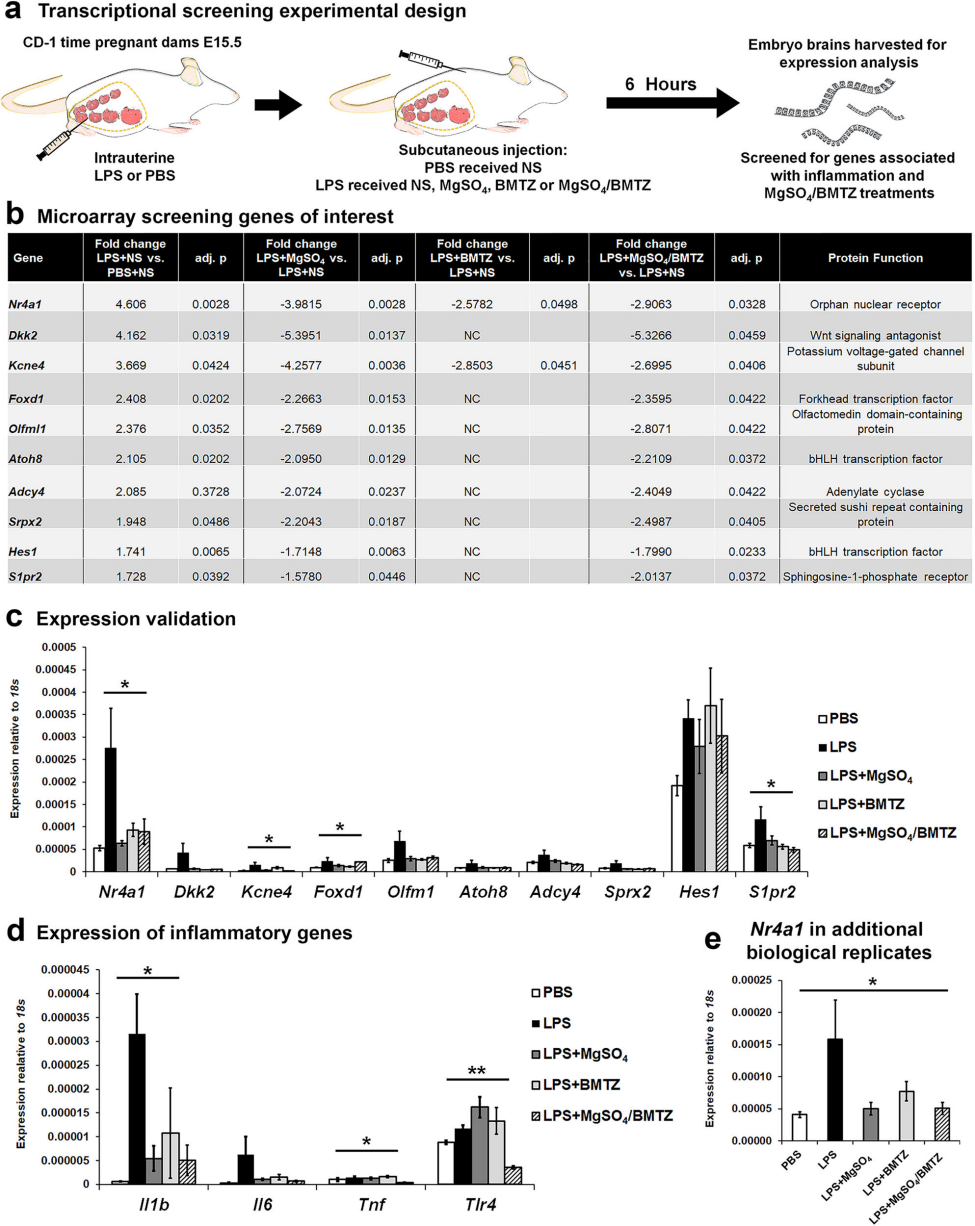
Screening for inflammatory regulators reveals the upregulation of *Nr4a1*. **a** CD-1 dams were randomized to receive an intrauterine injection of LPS or PBS at E15.5. Dams given LPS were randomized to receive subcutaneous injections of a combination of MgSO_4_ and/or BMTZ or the normal saline (NS) vehicle control. Mice that received PBS received NS. Brains were harvested for gene expression analysis 6 h following intrauterine injections. **b** The top genes upregulated with LPS and downregulated with treatment. Fold changes represent genes upregulated with LPS+NS groups vs. PBS+NS controls (column 2) and downregulated in LPS groups with MgSO_4_ (column 4), BMTZ (column 6), or the combination of MgSO_4_/BMTZ (column 8). Column 10 denotes a description of each gene. NC denotes no change. This comparison represents *N* = 4 embryo brains per condition, analyzed by microarray. **c** Validation by qRT-PCR in the same samples used for microarray analysis in **b**. **d** Expression of key inflammatory genes (*Il1b*, *Il6*, *Tnf* and *Tlr4*) by qRT-PCR, in the same samples assayed in **b**. **e** Additional embryo brains (total *N* = 8–9 per group) analyzed by qRT-PCR to confirm the upregulation of *Nr4a1*. *p < 0.05 and **p < 0.005 by one-way ANOVA. Error bars represent ±SEM.

To identify mediators of perinatal brain injury, we utilized microarray analysis to screen for inflammation-associated genes differentially expressed with LPS exposure and modulated by treatment with MgSO_4_, BMTZ, or the combination of MgSO_4_/BMTZ (Fig. 1a). The top ten genes upregulated with LPS injections and downregulated with at least one treatment were selected for further validation based on the degree of fold change and adjusted p-value. These genes included transcription factors (*Nr4a1*, *Foxd1*, *Atoh8*, *Hes1*), regulatory proteins (*Dkk2*, *Olfml1*, *Srpx2*, *Kcne4*), an enzyme (*Adcy4*) and a cell surface receptor (*S1pr2*) (Fig. 1b)^41–50^. Only *Nr4a1*, *Kcne4*, *Foxd1*, and *S1pr2* were significantly higher with LPS and similar to controls with treatments by qRT-PCR (Fig. 1c). The observed changes corresponded with the significant upregulation of proinflammatory genes: *Interleukin 1 beta* (*Il1b), Tumor necrosis factor* (*Tnf*), and *Toll-like receptor 4* (*Tlr4*) (Fig. 1d)^26,51^. Expression of *Interleukin 6* (*Il6*) was also elevated with LPS, although not significantly. In comparison to LPS alone, treatment with any combination of MgSO_4_ and/or BMTZ resulted in lower expression of these inflammatory factors. Interestingly, we observed an increase of *Tlr4* with LPS and either treatment administered individually. With the treatment combination, the expression of *Tlr4* was similar to PBS controls. From this screening and validation, we identified *Nr4a1* as the most prominent gene modulated by LPS and treatments. Validation in additional samples supported this result (Fig. 1e).

### *Nr4a1* KO mice show significant reductions in perinatal neuroinflammation and injury

**Fig. 2:**
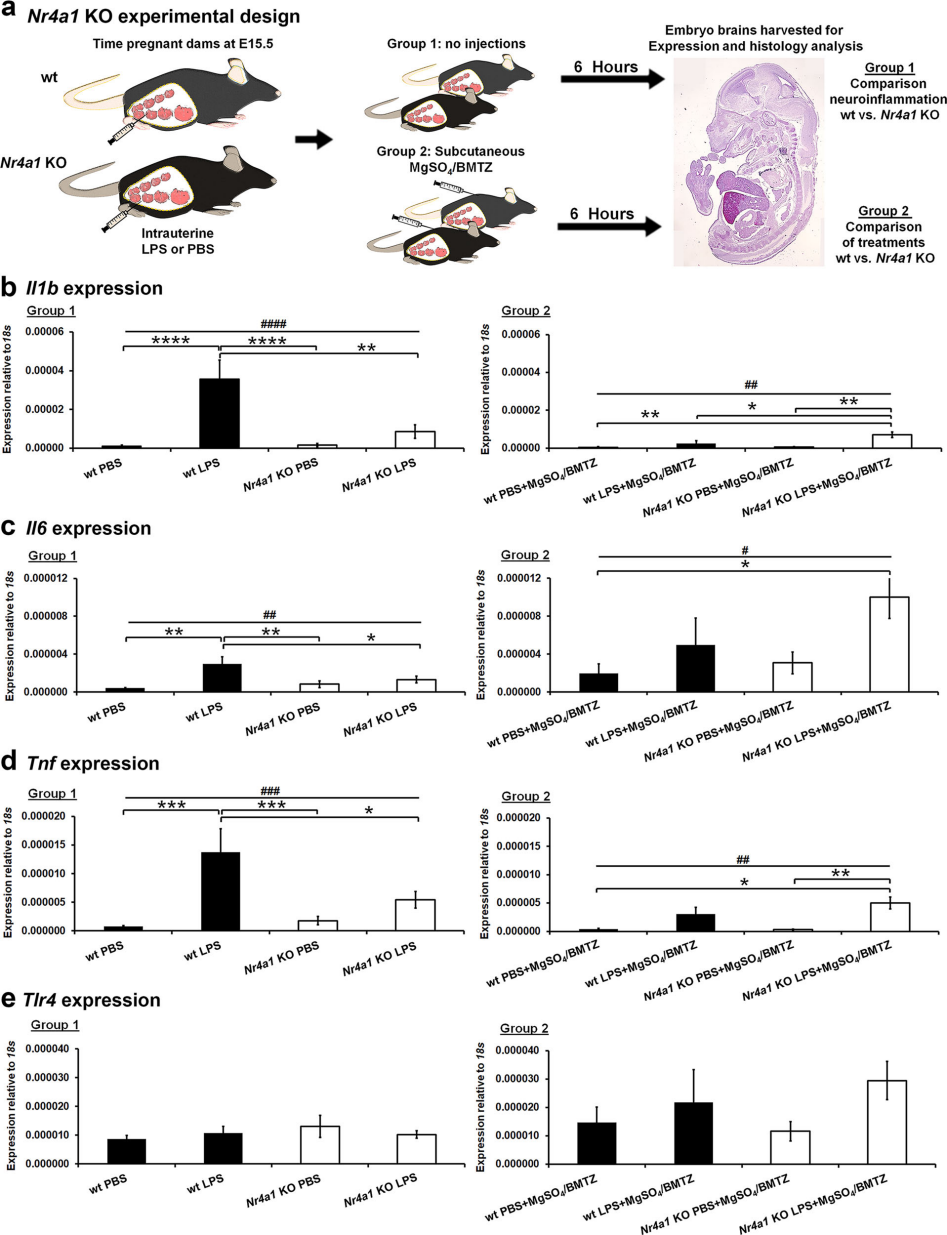
Examination of perinatal inflammation in the absence of *Nr4a1*. **a** Experimental design comparing wild type (wt) and *Nr4a1* knockout (KO) dams exposed to perinatal neuroinflammation at E15.5. Two groups of animals were evaluated; one comparing only PBS vs. LPS and a second comparing PBS vs. LPS with subsequent treatments of MgSO_4_/BMTZ. Embryo brains were harvested for molecular analysis, at 6 h following intrauterine injections of PBS or LPS. **b**–**e** Expression analysis for principal inflammatory genes *Il1b*, *Il6*, *Tnf*, and *Tlr4* was examined in E15.5 brains. This analyses represents per condition *N* = 13–17 brains for group 1 and *N* = 7–12 brains for group 2. ^#^*p* < 0.05, ^##^*p* < 0.005, ^###^*p* < 0.0005 by one-way ANOVA and *p < 0.05, **p < 0.005, ***p < 0.0005, ****p < 0.00005 by Tukey’s post-hoc test. Error bars represent ± SEM.

In order to assess whether *Nr4a1* regulates perinatal neuroinflammatory injury, we compared KO mice versus wt controls. Second, we compared LPS injury in wt and KO mice with the administration of MgSO_4_ and BMTZ to evaluate if the protective effects of these treatments are related to *Nr4a1*. The therapies of both MgSO_4_ and BMTZ were chosen due to their effectiveness when given in combination versus individual administration (Fig. 1d, e). These experiments were split into two groups, one evaluating LPS alone and a second comparing treatments. Group 1 consisted of wt and *Nr4a1* KO mice that received only LPS or PBS. Group 2 consisted of wt and *Nr4a1* KO mice that received LPS or PBS and MgSO_4_/BMTZ treatment (Fig. 2a). Although MgSO_4_ and BMTZ are relatively safe, their administration in PBS controls was included to account for any effects that may occur in mutant mice.

Once more, to evaluate the perinatal neuroinflammatory response to LPS we examined the expression of *Il1b, Il6, Tnf*, and *Tlr4* (Fig. 2b). Gene expression analysis by qRT-PCR revealed a robust upregulation of cytokines in wt versus *Nr4a1* KO fetal brains with LPS. The expression of *Il1b, Il6*, and *Tnf* in the wt brains was significantly elevated in the LPS group as compared to the PBS control (Fig. 2b, d, Group 1). In the *Nr4a1* KO brains, the expression of these cytokines was similar between PBS and LPS exposed animals. With the addition of MgSO_4_/BMTZ treatment, the wt inflammatory response was significantly lower than with LPS alone (Fig. 2b, d, Group 2). In contrast, the *Nr4a1* KO mice exposed to LPS showed greater expression of *Il1b* with LPS in comparison to PBS controls versus wt brains. Expression of *Il6* and *Tnf* cytokines was also higher in *Nr4a1* KO brains exposed to LPS and MgSO_4_/BMTZ, but only between certain treatment groups (Fig. 2c, d, Group 2).

**Fig. 3:**
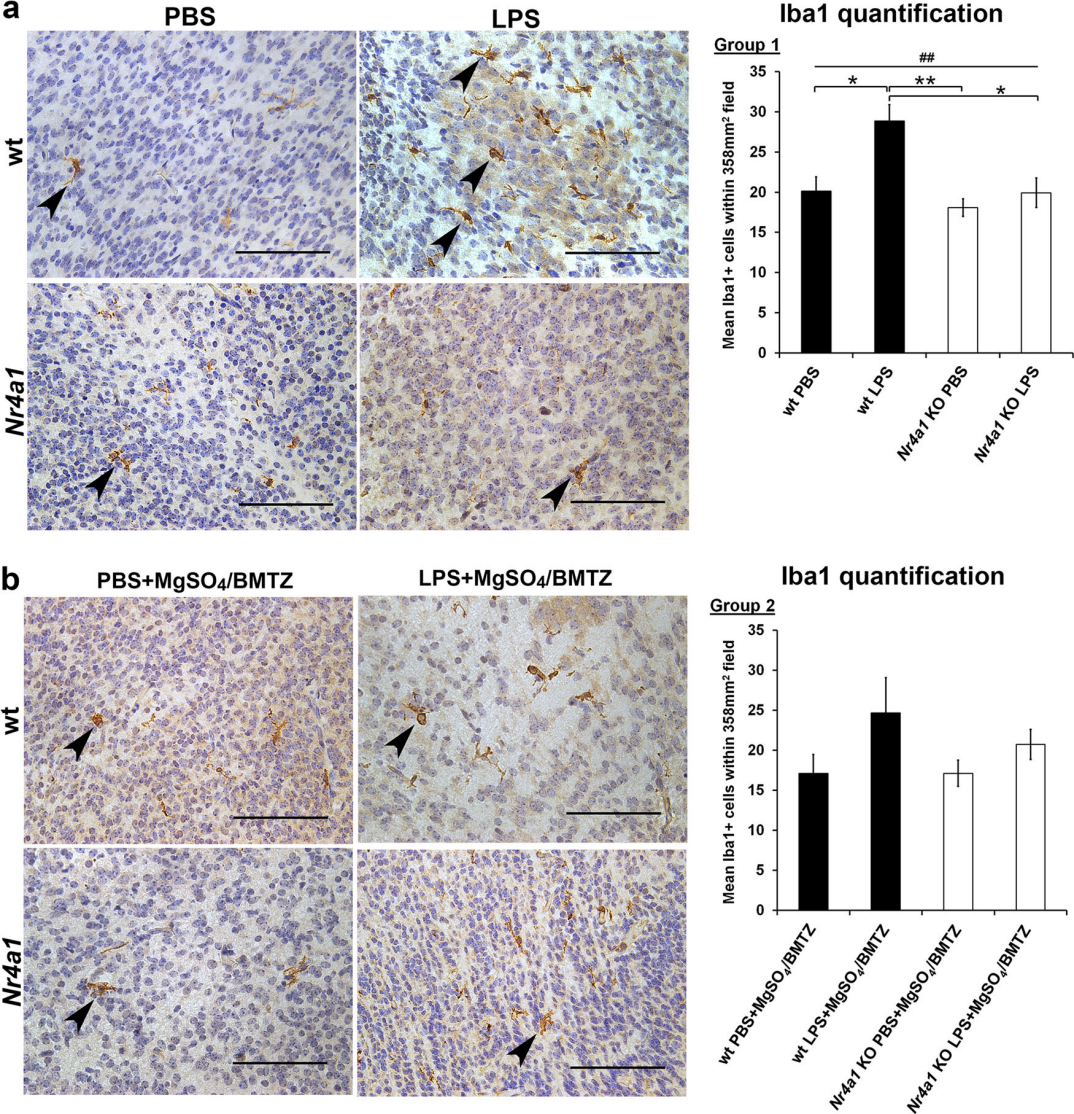
Microglial comparison between wt and *Nr4a1* KO embryos. E.15.5 brains from wt and *Nr4a1* KO embryos were stained for Iba1 with DAB (brown). Sections were counterstained with hematoxylin to visualize nuclei in blue and high magnification micrographs (40×) depict Iba1+ microglia (arrowheads) between conditions. Cell quantifications graphed to the right were conducted at a lower magnification (20×) in order to reduce error. **a** Comparison and quantification of Iba1+ cells for group 1 (*N* = 6 brains per condition); wt and *Nr4a1* KO animals were exposed to PBS or LPS. **b** Comparison and quantification for group 2 (*N* = 3 brains per condition); wt and *Nr4a1* KO animals were exposed to PBS or LPS and MgSO_4_/BMTZ treatment. Scale bars denote 100 µm. ^##^*p* < 0.005 by one-way ANOVA and *p < 0.05, **p < 0.005, by Tukey’s post-hoc test. Error bars represent ± SEM.

In addition to gene expression, we also compared the cellular response to neuroinflammation by quantifying the number of Iba1+ microglia. With LPS, a greater number of Iba1+ cells was observed in wt embryo brains (Fig. 3a)^52,53^. No difference was observed in the brains of *Nr4a1* KO embryos with PBS or LPS exposure. With MgSO_4_/BMTZ treatment, the number of Iba1+ cells in wt brains was higher but not significantly with LPS vs. PBS controls (Fig. 3b). There was also no significant difference in *Nr4a1* KO brains with MgSO_4_/BMTZ treatment.

**Fig. 4:**
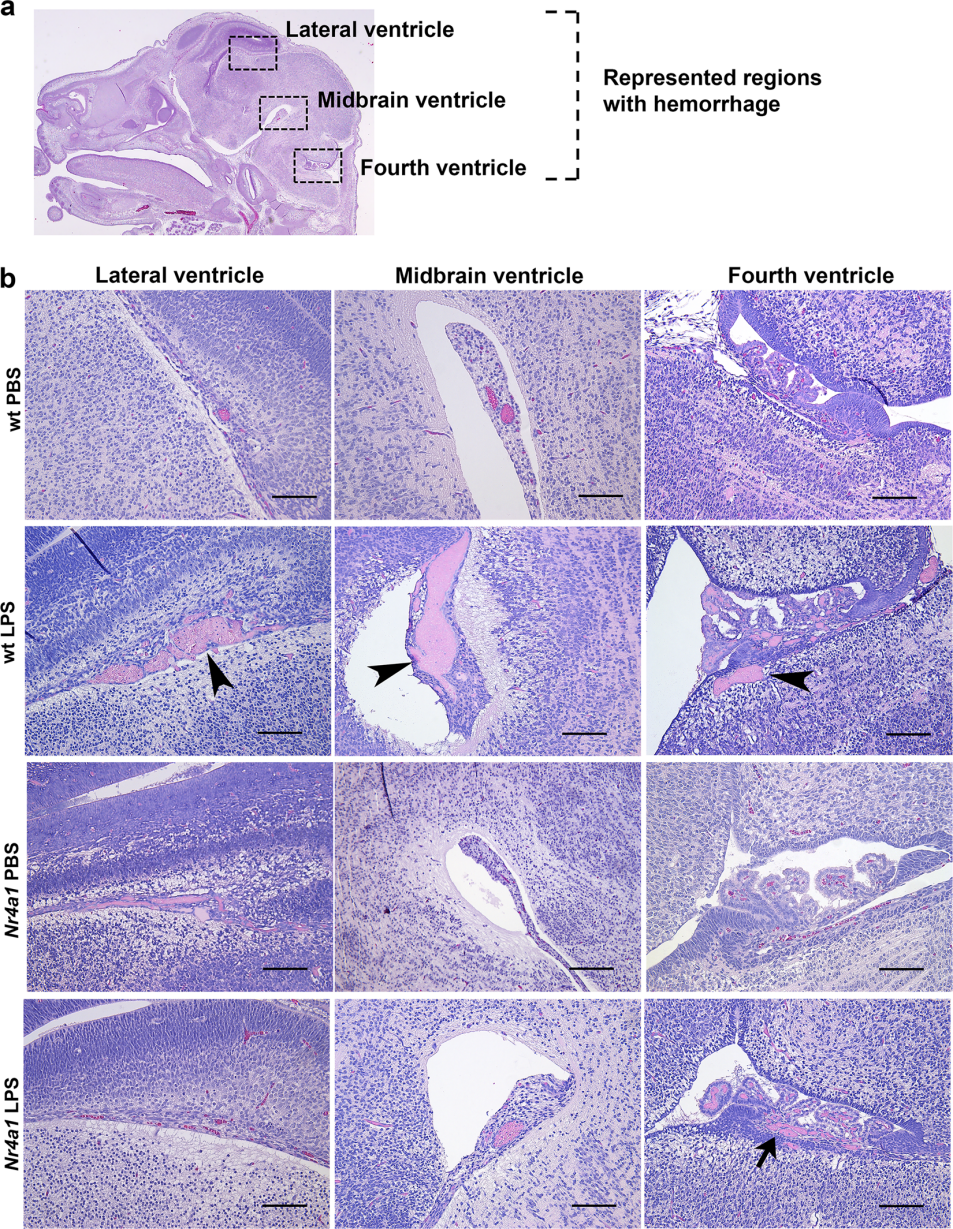
Comparison of cerebral pathology observed in wt and *Nr4a1* KO embryos. **a** H&E stained cross-section of an E15.5 brain, highlighting the regions (boxed areas) where injury in the form of hemorrhage was mainly observed. **b** Representative micrographs of hemorrhage in the respective areas described in **a** for group 1 (PBS vs. LPS). Hemorrhage was predominant in wt brains exposed to LPS (arrowheads) and to a lesser extent in *Nr4a1* KO brains also exposed to LPS (arrow). Scale bars denote 100 µm.

**Fig. 5:**
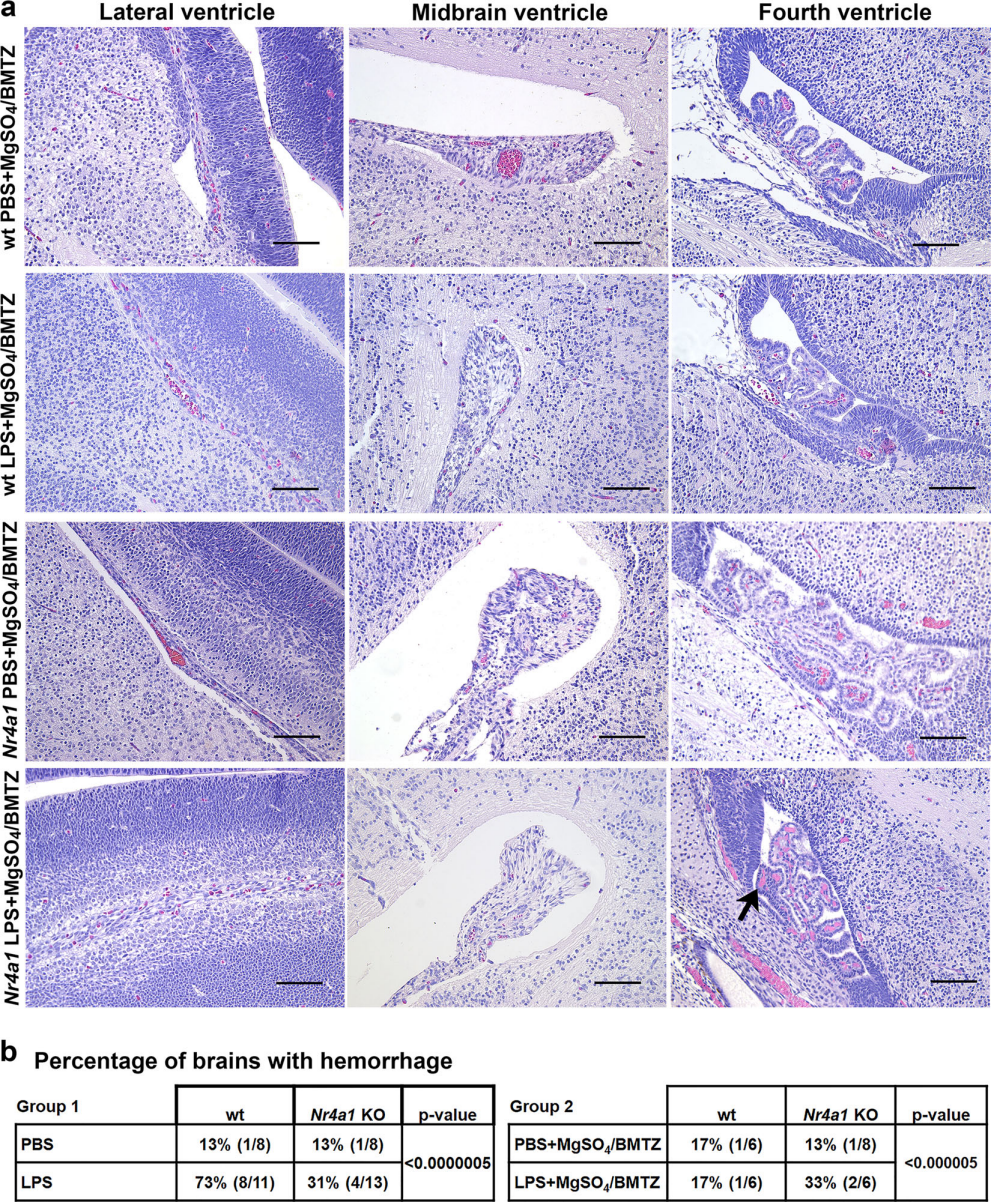
Reductions of cerebral pathology in *Nr4a1* KO embryos. **a** Representative micrographs of hemorrhage (arrow), in respective areas for group 2 (PBS vs. LPS with MgSO_4_/BMTZ). Scale bars denote 100 µm. **b** The frequency of cerebral hemorrhage observed per condition for groups 1 and 2. The number of brains with hemorrhage vs. total examined is indicated in the brackets to the right of the percentages. *P*-values were generated by chi-squared test using the PBS or PBS + MgSO_4_/BMTZ controls as a reference.

To gain more insight into the role of *Nr4a1* in perinatal brain injury, we examined the histopathology in wt versus mutant embryo brains. Most obvious was cerebral hemorrhage, occurring predominantly in the lateral ventricle, midbrain, and fourth ventricle (Fig. 4a). This pathology was more pronounced in wt animals exposed to LPS in comparison to PBS controls and *Nr4a1* KO brains (Fig. 4b). The frequency of hemorrhage in wt mice exposed to LPS diminished with MgSO_4_/BMTZ treatment (Fig. 5a). The frequency of hemorrhage was also significantly different between wt and mutant animals in both Groups 1 and 2 (Fig. 5b). Notably, the incidence of hemorrhage in *Nr4a1* KO brains exposed to LPS and MgSO_4_/BMTZ was greater versus wt counterparts and similar to mutant mice that received only LPS.

### *Nr4a1* is upregulated by cerebral endothelial cells in response to neuroinflammation

**Fig. 6:**
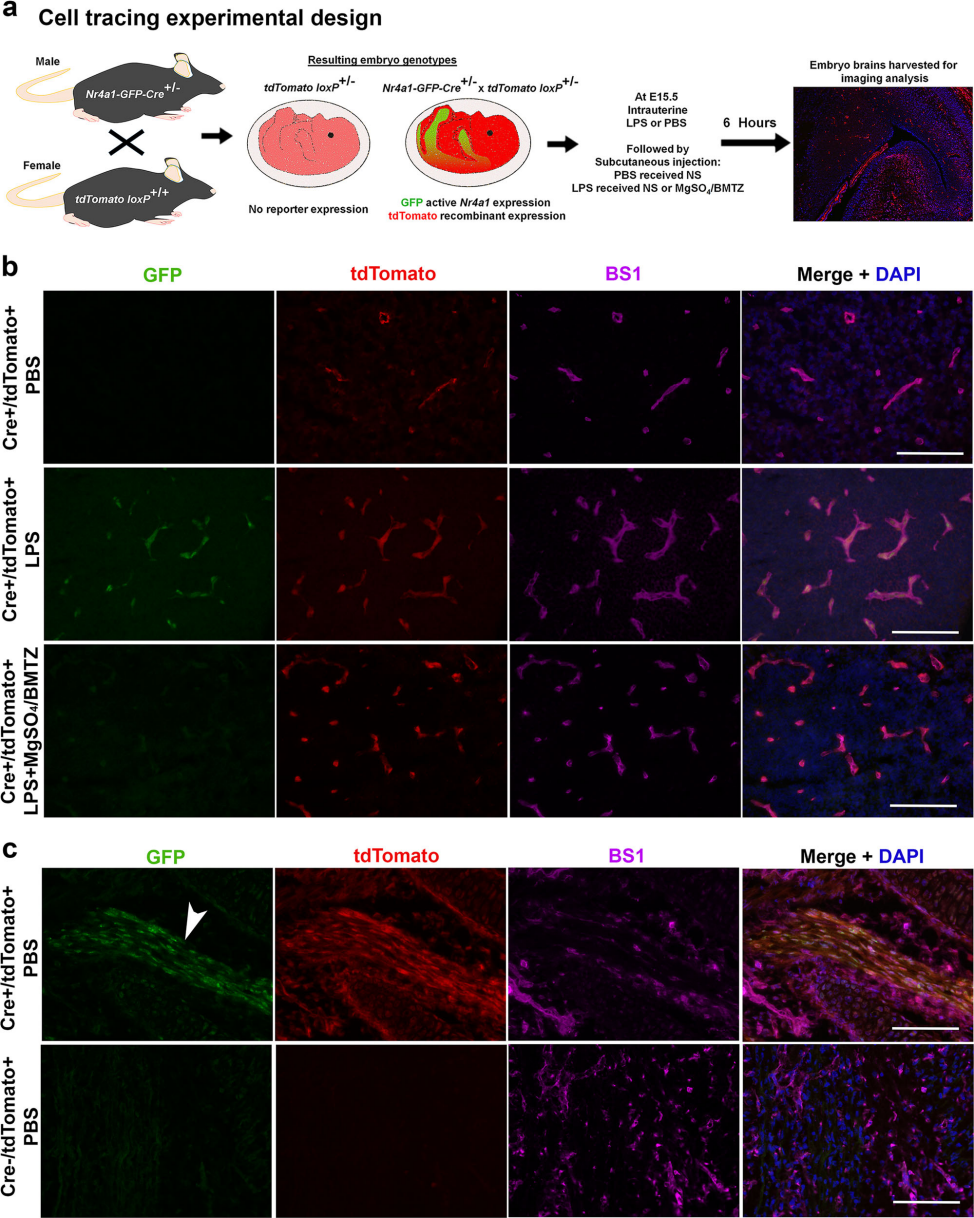
Fate mapping *Nr4a1* expression with perinatal neuroinflammation. **a** Experimental design for combining the *Nr4a1-GFP-Cre* and *tdTomato loxP* alleles in E15.5 embryos. Males heterozygous for *Nr4a1-GFP-Cre* were timed mated with females homozygous *for tdTomato loxP* to generate embryos that harbor both alleles at a 50% Mendelian frequency. For this comparison, *tdTomato loxP* mice were given intrauterine PBS and subcutaneous NS, LPS and NS or LPS and MgSO_4_/BMTZ. Embryo brains were harvested 6 h following injections for fluorescence microscopy. **b** Representative micrographs for each genotype portraying GFP (green), indicative of real-time *Nr4a1* expression, and tdTomato (red), expressed irreversibly following Cre recombination. BS1 (magenta) was used to label the vasculature. Merged images combine all three channels with DAPI (blue). **c** Positive (skeletal muscle, arrowhead) and negative controls (Cre negative littermates) confirm the specificity of the Cre-loxP combination represented in (**a**). Scale bars denote 100 µm.

To characterize the cellular expression of *Nr4a1*, we utilized Cre-loxP fate mapping with the *Nr4a1*-*GFP-Cre* model. In addition to Cre, this allele expresses GFP as an indicator of active *Nr4a1* expression. To compare GFP and Cre labeling, *tdTomato loxP* females were mated with *Nr4a1*-*GFP-Cre* males and given intrauterine PBS, LPS or LPS and MgSO_4_/BMTZ at E15.5 (Fig. 6a). In brains of PBS controls, we observed tdTomato localized to the vasculature (Fig. 6b). LPS exposed animals had tdTomato and GFP, both appearing localized to the cerebral vasculature. Analogous to PBS controls, we observed only tdTomato in brains exposed to LPS and MgSO_4_/BMTZ. Staining with the lectin BS1 confirmed that GFP and tdTomato reporters localized to the vasculature^31^. Both GFP and tdTomato reporters were present in the developing skeletal muscle (Fig. 6c), corresponding to the known expression of *Nr4a1* in myoblasts^54^. Concurrently, fluorescence for either reporter was absent in littermates negative for the *Nr4a1*-*GFP-Cre* allele.

**Fig. 7:**
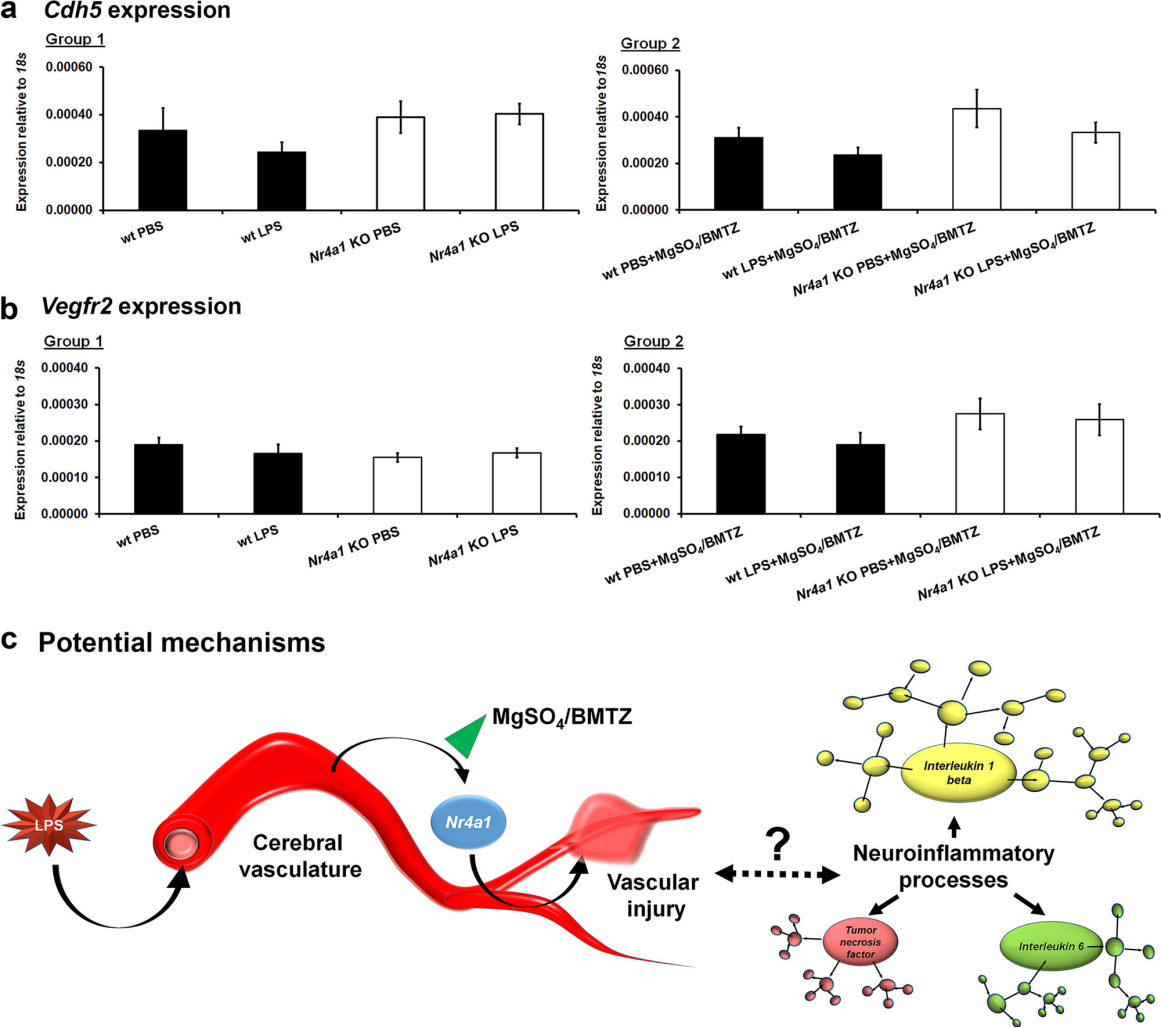
Vascular gene expression in *Nr4a1* KO embryos and summary of findings. Embryonic brains from Fig. 2 were evaluated for the expression of **a** *Cdh5* and **b** *Vegfr2*. No statistical significance by was noted by one-way ANOVA between wt and *Nr4a1* KO animals or with MgSO_4_/BMTZ treatment. Error bars represent ±SEM. **c** Through undetermined interactions the expression of *Nr4a1* with perinatal neuroinflammation results in vascular injury (hemorrhage). In turn, the neuroinflammatory process is activated directly or indirectly, as indicated by the upregulation of principal cytokines *Il1b*, *Il6*, and *Tnf*. This process appears blunted by MgSO_4_/BMTZ, which may be related to *Nr4a1* suppression.

Based on the pattern of cellular expression we examined *Nr4a1* KO versus wt brains (same samples used in Fig. 2) for differences in vascular genes, *Cdhr5* and *Vegfr2*. Both genes are important for endothelial integrity and angiogenesis. Therefore a difference from PBS controls may reflect intrinsic differences in vascular development that may influence the outcomes observed in *Nr4a1* KO brains with LPS exposure^55,56^. Secondarily, we sought to gain more insight into possible declines in endothelial cell integrity or number that may also occur with LPS. Expression analysis indicates no such differences even with MgSO_4_/BMTZ (Fig. 7a, b).

## Discussion

We found that *Nr4a1* is upregulated with neuroinflammation in a murine model of preterm labor. Treatments with MgSO_4_/BMTZ mitigated this response in wt animals. In contrast, *Nr4a1* KO animals showed a reduction in neuroinflammation and brain injury. MgSO_4_/BMTZ did not alter inflammatory responses in mutant animals. Finally, using Cre-loxP fate mapping, we were able to identify that the inflammatory upregulation of *Nr4a1* occurs in the vasculature of the fetal brain.

With normal brain development, *Nr4a1* is limited or absent according to several lines of evidence^21,22^. In situ hybridization of fetal mouse brains show an absence of *Nr4a1* until E18.5 (© 2015 Allen Institute for Brain Science, Allen Brain Atlas API; available from: http://developingmouse.brain-map.org/gene/show/15145). Within the context of preterm birth, *Nr4a1* is upregulated with lung inflammation in premature sheep^57^. In addition, microarray analysis of fetal rat brains 4 h following LPS exposure reveals an increase of *Nr4a1* expression (GEO accession GDS4429)^23^. This coincides with the significant increase of *Nr4a1* that we observed at 6 h following LPS. Based on this pattern of upregulation with insult, downregulation with MgSO_4_/BMTZ treatments, and limited presence in brain development, we reasoned that *Nr4a1* warranted further investigation. We took a genetic approach and used an established mutant mouse model with no known developmental phenotype^22^. The lack of differences between PBS controls (Figs. 2–5, 7a, b) support the notion that *Nr4a1* ablation does not influence normal development. However, with the addition of an inflammatory instigator there are significant changes that may cause neurodevelopmental deficiencies noted in postnatal animals^36^.

Our study suggest *Nr4a1* plays a prominent role in perinatal neuroinflammation and brain injury. Conversely, *Nr4a1* has been implicated in both anti- and pro-inflammatory processes. Within the adult innate immune system and with metabolic disease, *Nr4a1* plays an anti-inflammatory role^58,59^. The mechanisms by which *Nr4a1* reduces inflammation have been noted to involve p38, NF-kB, and ISG12 depending on the context of disease, biological modeling, and analyses^19,60,61^. In- depth molecular examination suggests p38 counters *Nr4a1* in suppressing NF-kB pro-inflammatory signaling^19^. Whereas comparisons of animal models reveals *Nr4a1* deficient mutants exhibit greater inflammation in response to sepsis and higher mortality versus *Isg12* deficient animals^61^. In the adult CNS, *Nr4a1* is broadly expressed in brain tissue and involved in regulatory functions, specifically in microglia. In contrast to our results in embryonic brains, adult *Nr4a1* KO mice show elevated autoimmune inflammatory responses by microglia and T-cells^62–64^.

In addition to the anti-inflammatory responses, there are numerous biological processes whereby *Nr4a1* is pro-inflammatory. Nr4a receptors are expressed in several immune cells to include activated macrophages^20^. Within the context of LPS stimulation, *Nr4a1* can also promote macrophage NF-kB signaling^65^. Other studies have indicated that *Nr4a1* does not alter immune cell responses but instead antagonizes endothelial responses to inflammation^66^. In regards to the CNS, *Nr4a1* has been implicated in adult brain injury to promote neuroinflammation and cell death^17,18^. Although these studies did not link the cerebral vasculature to *Nr4a1* signaling, it is important to note that trauma was modeled by inducing subarachnoid hemorrhage. In regard to a role within the vasculature, various modalities have implicated *Nr4a1* in regulating endothelial cell inflammation, leakiness, permeability and dysfunction^40,66–68^. Such studies reinforce that *Nr4a1* is dispensable for homeostasis but is important in a pathological state. This pattern of *Nr4a1* necessity makes it an ideal target for disease, since ablation in normal processes may not result in deleterious side effects.

In this study, the absence of *Nr4a1* in KO mice resulted in a significant reduction of principal pro-inflammatory markers in the E15.5 pup brains. This evidence provides a basis for the role of *Nr4a1* in regulating bacteria initiated perinatal neuroinflammation. It is possible that *Nr4a1* promotes an increase in cytokine expression from an inflammatory insult, as displayed by the effect of LPS in the wt mice. In comparing the LPS treatment groups of the wt and the *Nr4a1* KO, the treatment of MgSO_4_/BMTZ did not appear to decrease the level of inflammatory cytokine expression. This may be explained by a potential mechanism related to *Nr4a1* signaling that is essential for the neuroprotective actions of MgSO_4_/BMTZ. Alternatively, in *Nr4a1* KO animals inflammation may be reduced to a threshold whereby the treatments are no longer effective. We additionally recognize that by using MgSO_4_/BMTZ in combination (a clinically relevant approach), our results cannot be attributed to one of the two medications.

The upregulation of pro-inflammatory genes with LPS exposure corresponds with a greater number of microglia in wt brains. Treatment with MgSO_4_/BMTZ mitigated the increase of microglia in wt embryos that was observed with LPS alone. In mutant embryos exposed to LPS, the number of microglia did not elevate or correlate with increases in pro-inflammatory gene expression. In response to inflammatory stimuli such as LPS, microglia produce cytokines including IL-1β^69^. It is possible that the microglial inflammatory response is restricted in mutant embryos, resulting in significantly lower levels of cytokine expression vs. wt animals. Alternatively, other cell population’s upregulate cytokines independent of the mutant status but to a limited degree as compared to microglia.

To further characterize the role of *Nr4a1* in perinatal brain injury, we examined the histopathology of the embryo brains. Cerebral hemorrhage was notable in wt versus *Nr4a1* KO brains with LPS. MgSO_4_/BMTZ mitigated the frequency of hemorrhage in wt brains but had no effect in KO animals. These results correspond with the pathological findings in humans, whereby the incidence of intraventricular hemorrhage is greater with prematurity and reduced by corticosteroids^70–72^. The mechanisms of perinatal hemorrhage within the context of preterm labor are not clear. Our examination of key vascular genes suggests that cerebral hemorrhage is not a result of endothelial cell decline (Fig. 7a, b). Both MgSO_4_ and corticosteroids are known to relax the vasculature^73,74^. However, it is unclear whether vasodilation or other responses invoked by MgSO_4_ and/or BMTZ confer neuroprotection. Our results suggest a possible role of *Nr4a1* with regard to MgSO_4_/BMTZ and the cerebral vasculature. This is supported by the upregulation of *Nr4a1* in vessels observed with Cre-loxP fate mapping.

Whether *Nr4a1* signaling directly or indirectly influences the perinatal inflammatory response remains a question. Given that *Nr4a1* is expressed in the vasculature, we suspect that it is mediating inflammation as summarized in Fig. 7c. One of the principal inflammatory markers, *Il1b*, was intimately affected by the knockout status and the treatment group. This suggests that *Il1b* expression is modulated by *Nr4a1*. However, it is unclear whether the upregulation of inflammatory cytokines by *Nr4a1* is direct or indirect. Vascular cells are known to express interleukin receptors and respond to inflammatory cytokines^75^. Therefore, it is likely that perinatal vascular cells interact with immune cells, but are not the main source of inflammatory cytokines.

Our study’s strengths include the use of multiple murine models including outbred CD1, inbred C57BL/6 and *Nr4a1* KO mice, to support our findings. Furthermore, several assays and validation experiments were conducted including the use of Cre-loxP mutants that not only substantiated the upregulation of *Nr4a1* with neuroinflammation but also implicated the vasculature. Finally, the comparison of MgSO_4_/BMTZ provides additional insight into the mechanisms of neuroprotection and the role of *Nr4a1*.

The primary limitation of our study is the reliance on a mouse model of preterm labor. The mechanisms of preterm labor in humans are complex and difficult to translate. Therefore, we used an inflammation-based model which has been well described in the literature. Although other exposure modalities using live bacteria exist, we used the *in utero* LPS approach to examine specific inflammatory components of brain injury to evaluate potential mechanisms of MgSO_4_/BMTZ neuroprotection^2,51,76^.

A second limitation is the use of the microarray only for screening genes but not for functional analysis. This is due to the low number of genes that were validated; only four of our top ten were significant by qRT-PCR (Fig. 1c). Based on the high level of false positives, we deemed the microarray unsuitable for making interpretations regarding the status of the transcriptome.

A third limitation is that we could not establish a clear link between *Nr4a1* and its mechanism of action. We suspect that the mechanisms of *Nr4a1* are directly related to the vasculature and the pathology of hemorrhage. However, we could not pinpoint the role of *Nr4a1* to specific pathways within the neuroinflammatory milieu. We observed that *Nr4a1* is re-expressed in the context of neuroinflammation but the exact timing and reason for expression earlier in development also remain unclear. Finally, the etiology of preterm labor in humans is multifactorial and our observations may represent only one of many pathways that govern neuroinflammation and/or MgSO_4_/BMTZ neuroprotection^77^.

In closing, we have identified *Nr4a1* as a potent mediator of perinatal neuroinflammation and direct or indirect target of MgSO_4_/BMTZ treatments. *Nr4a1* expression was upregulated in the fetal brain vasculature and linked to cerebral hemorrhage. The lack of *Nr4a1* in normal brains presents an opportunity to target neuroinflammation, potentially with few developmental side effects. Based on these findings, additional studies are warranted to better understand the role of the vasculature and *Nr4a1* signaling within the context of preterm labor.

## Supplementary information

Supplementary Fig S1
