# Correction: The orphan nuclear receptor *Nr4a1* mediates perinatal neuroinflammation in a murine model of preterm labor

**DOI:** 10.1038/s41419-020-2682-y

**Published:** 2020-06-30

**Authors:** Sarah M. Estrada, Andrew S. Thagard, Mary J. Dehart, Jennifer R. Damicis, Elisabeth M. Dornisch, Danielle L. Ippolito, Irina Burd, Peter G. Napolitano, Nicholas Ieronimakis

**Affiliations:** 1 Department of Obstetrics and Gynecology, Division of Maternal Fetal Medicine, Madigan Army Medical Center, Tacoma, WA USA; 2 Department of Clinical Investigation, Madigan Army Medical Center, Tacoma, WA USA; 3 PharmaWrite Medical Communications LLC, Princeton, NJ USA; 4 Integrated Research Center for Fetal Medicine, Johns Hopkins University School of Medicine, Baltimore, MD USA; 5 Department of Obstetrics and Gynecology, University of Washington Medical Center, Seattle, WA USA

Correction to: *Cell Death & Disease*

10.1038/s41419-019-2196-7 published online 06 January 2020

This Article was originally published with the incorrect copyright line “The Authors”. The correct copyright is “US Govt”.

The PDF and HTML versions of the Article have been modified accordingly.

