# Supplementary material for: The orphan nuclear receptor Nr4a1 mediates perinatal neuroinflammation in a murine model of preterm labor
Source: Cell Death Dis. 2020 Jan 6;11(1):11. doi: 10.1038/s41419-019-2196-7 (PMC6944691; doi:10.1038/s41419-019-2196-7)
Supplement: Supplementary file 1 — Supplementary Fig S1 [file 41419_2019_2196_MOESM1_ESM.docx]

| **Table S1: qRT-PCR primer sequences** | | | |
| --- | --- | --- | --- |
| **Gene** | **Forward** | **Reverse** | **Size (bp)** |
| *18s* | TTGACGGAAGGGCACCACCAG | GCACCACCACCCACGGAATCG | 120 |
| *Nr4a1* | TTGAGTTCGGCAAGCCTACC | GTGTACCCGTCCATGAAGGTG | 100 |
| *Dkk2* | CTGATGCGGGTCAAGGATTCA | CTCCCCTCCTAGAGAGGACTT | 126 |
| *Foxd1* | GGGAGAGCGAAGGTAGGACTC | GTTGCAGCATAGGGCGATTT | 205 |
| *Olfml1* | GGTGTCAGACCCACACAAGT | GTCACAGCTTGTGTTCAACAGGG | 209 |
| *Atoh8* | TGGAAGACTGTGTGCGTTAAAG | TCCAAGTCCAATCGGAAAGTTTT | 110 |
| *Adcy4* | GTCCTTGGACTGTATCTTGGGT | CCACACAGGAACAATACCGC | 89 |
| *Srpx2* | ATGGTACGCAGGCTCAGGTTA | TGAGTAGCATGTGGCTTCTCC | 154 |
| *Hes1* | CCAGCCAGTGTCAACACGA | AATGCCGGGAGCTATCTTTCT | 166 |
| *S1pr2* | AGTGACAAAAGCTGCCGAATG | GCACGTAGTGCTTAGCATAGAGAGG | 151 |
| *Il1b* | GAAATGCCACCTTTTGACAGTG | TGGATGCTCTCATCAGGACAG | 116 |
| *Il6* | CTGCAAGAGACTTCCATCCAG | AGTGGTATAGACAGGTCTGTTGG | 131 |
| *Tnf* | CCTGTAGCCCACGTCGTAG | GGGAGTAGACAAGGTACAACCC | 148 |
| *Tlr4* | GCCTTTCAGGGAATTAAGCTCC | GATCAACCGATGGACGTGTAAA | 114 |
| *Cdhr5* | GTCGATGCTAACACAGGGAATG | AATACCTGGTGCGAAAACACA | 185 |
| *Vegfr2* | TTTGGCAAATACAACCCTTCAGA | GCAGAAGATACTGTCACCACC | 133 |
